# The Effect of Ionic Soil Stabilizer on Cement and Cement-Stabilized Iron Tailings Soil: Hydration Difference and Mechanical Properties

**DOI:** 10.3390/ma18071444

**Published:** 2025-03-25

**Authors:** Hongtu Li, Jian Jia, Xiaolei Lu, Xin Cheng, Jiang Zhu, Lina Zhang, Peipei Guo, Gongning Zhai

**Affiliations:** 1 Shandon Provincial Key Laboratory of Preparation and Measurement of Building Materials, University of Jinan, Jinan 250022, China; hongtu-li@foxmail.com (H.L.); mse_luxl@ujn.edu.cn (X.L.); mse_zhuj@ujn.edu.cn (J.Z.); mse_zhangln@ujn.edu.cn (L.Z.); 2 School of Materials Science and Engineering, University of Jinan, Jinan 250022, China; 3 Qingdao Highway Development Center, Qingdao 266075, China; jiajianxml@foxmail.com (P.G.); qdgljhc@qd.shandong.cn (G.Z.)

**Keywords:** ionic soil stabilizer, cement hydration, cement-stabilized iron tailings soil, mechanical properties

## Abstract

The ionic soil stabilizer (ISS) can synergistically enhance the mechanical properties and improve the engineering characteristics of iron tailings soil in conjunction with cementitious materials such as cement. In this paper, the influence of ISS on the cement hydration process and the charge repulsion between iron tailings soil particles was studied. By means of Isothermal calorimetry, X-ray diffraction (XRD), Scanning electron microscope (SEM), and Low-field nuclear magnetic resonance microscopic analysis methods such as (LF-NMR), X-ray photoelectron spectroscopy (XPS), Non-evaporable water content and Zeta potential were used to clarify the mechanism of ISS-enhanced cement stabilization of the mechanical properties of iron tailings soil. The results show that in the cement system, ISS weakens the mechanical properties of cement mortar. When ISS content is 1.67%, the 7 d compressive strength of cement mortar decreases by 59.8% compared with the reference group. This retardation arises due to carboxyl in ISS forming complexes with Ca^2+^, creating a barrier on cement particle surfaces, hindering the hydration reaction of the cement. In the cement-stabilized iron tailings soil system, ISS has a positive modification effect. At 0.33% ISS, compared with the reference group, the maximum dry density of the samples increased by 6.5%, the 7 d unconfined compressive strength increased by 35.3%, and the porosity decreased from 13.58% to 11.85%. This is because ISS reduces the double electric layer structure on the surface of iron tailings soil particles, reduces the electrostatic repulsion between particles, and increases the compactness of cement-stabilized iron tailings soil. In addition, the contact area between cement particles increases, the reaction energy barrier height decreases, the formation of Ca(COOH)_2_ reduces, and the retarding effect on hydration weakens. Consequently, ISS exerts a beneficial effect on augmenting the mechanical performance of cement-stabilized iron tailings soil.

## 1. Introduction

Traditional calcium-based stabilizers are the most widely used for soil stabilization [1,2,3]. However, the use of lime and cement to treat soil containing sulfate will lead to an increase in volume change, and water stability cannot meet the needs of engineering construction [4]. Additionally, stabilizing soils by cement and lime may cause dry reflective cracks [5].

The ionic soil stabilizer (ISS) represents a non-traditional stabilizer for expansive soils supplied in the form of a concentrated liquid, which is also utilized for the treatment of tailings possessing soil-like properties. It is common practice to dilute it with water in a specific proportion and mix it thoroughly with the soil before compacting the soil on site [6,7]. ISS can effectively mitigate the swelling behavior and increase the unconfined compressive strength (UCS) of expansive soil [8,9]. The synergistic stabilization of ISS and calcium-based stabilizers has been suggested as effective measures to improve soil strength and reduce swelling behavior [10]. ISS is a liquid chemical reagent composed mainly of electrolytes and enzymes, which are composed of common cationic groups such as ammonium, calcium, and magnesium ions, as well as anions including phosphate, carboxyl, chloride, and bromide [11,12]. There are differences in the composition of the ISS depending on the manufacturer. However, most of the ISS currently used to solidify soil works through the encapsulation of clay minerals, exchange of interlayer cations, breakdown of clay minerals with expulsion of water from the double layer, and interlayer expulsion with subsequent moisture entrapment [13,14].

The combination of ISS and cement can be used to stabilize soil/tailings, improving its strength and water stability while reducing reflective cracks [15,16]. This method is effective in compensating for the deficiencies of using a single material for solidification [17]. Chen et al. [18] investigated the hydrophobic ionic soil stabilizer, that is, the synergistic effect of the hydrophobic ionic curing agent and cement on the improvement of loess performance, ISS makes more water participate in the hydration reaction. In addition, ISS ions replace the cations on the surface of the loess particles, promoting flocculation and agglomeration between soil particles, which enhances the soil water stability [6,14,19]. Ma et al. [20] used Portland cement and ISS to treat organic-contaminated soil, which produced a 2.3-fold increase in UCS and a significant improvement in the freeze-thaw resistance of the solidified body. This was attributed to the fact that Ca^2+^ in ISS replaced monovalent cations in the soil to reduce the thickness of the double layer of water and increase the attraction between particles.

Existing studies have preliminarily verified the synergistic effect of ISS and calcium-based stabilizers, but the micro-mechanism of their interaction is still lacking multi-scale characterization. In this regard, this paper systematically studied the effects of ISS on cement hydration and surface charge repulsion of tailings particles by means of various characterization methods. It aimed to clarify the mechanism of ISS improving the mechanical properties of cement-stabilized iron tailings soil, so as to better understand the synergic solidification effect of cement on iron tailings soil and find an effective, environmentally friendly, and economic solution for iron tailings soil improvement.

## 2. Materials and Experimental Methods

### 2.1. Materials

#### 2.1.1. ISS

ISS used in the test was purchased from Beijing Oriental Yuhong Sand Powder Technology Co., Ltd. (Beijing, China). In this study, ISS was employed to enhance the mechanical properties of cement-stabilized iron tailings soil. The ISS was synthetically formulated within the laboratory environment. The solution contained a notable quantity of Al^3+^, Ca^2+^ and Na^+^ ions, and maintained a pH range of 6.5 to 7.0. This ISS exhibited favorable solubility in deionized water and possessed a density of 1.13 g/cm^3^. ISS is capable of disrupting the electrical double layer structure composed of Na^+^ and K^+^ ions on the surface of iron tailings soil particles, enabling a more compact arrangement under equivalent compaction energy [15,21]. Consequently, this enhances the mechanical properties of the iron tailings soil.

#### 2.1.2. Cement

The cement used in this experiment was purchased from P.O42.5 cement produced by Jinan Shanshui cement factory, Shandong Province. The basic performance indexes of cement are shown in Table 1. Its chemical composition is determined by X-ray fluorescence (XRF) spectroscopy, as shown in Table 2.

#### 2.1.3. Iron Tailings Soil

**Figure 1:**
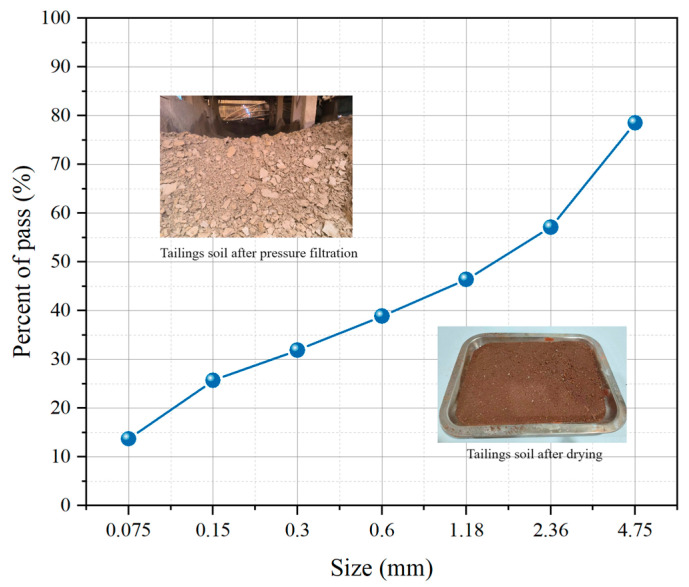
The particle size distribution of iron tailings soil.

In the present investigation, the iron tailings soil is taken from Qingdao City, Shandong Province. According to the JTG 3430-2020 [22] normative guidelines, the iron tailings soil exhibited a plastic limit of 14.6% and a liquid limit of 27.8%. The plasticity index, measured as 13.2, falls within the classification of silty clay as defined in the standard GB/T 50547-2022 [23]. The particle size distribution of the dried iron tailings soil was illustrated in Figure 1, and its specific chemical composition is shown in Table 3.

### 2.2. Mix Proportion and Sample Preparation

#### 2.2.1. Experiment of ISS on Cement Hydration

Table 4 outlines the mixing ratios of ISS and cement, with a fixed water-to-cement ratio of 0.5. ISS dissolved in mixing water, proportionally to cement mass, at ratios (ISS/cement) of 0.17%, 0.33%, and 1.67%, deviating from standard cement admixture practice. Resultant cement pastes are designated as ISS0, ISS017, ISS033, and ISS167. The cement used is P.O42.5 cement produced by Jinan Shanshui cement factory, and the sand used is standard sand, which meets the requirements of Chinese ISO standard sand and contains three levels of composition. Rough sand is 1.0 mm–2.0 mm, medium sand is 0.50 mm–1.0 mm, fine sand is 0.08 mm–0.50 mm, and the content of SiO_2_ is >97%.

#### 2.2.2. Experiment of ISS on Cement-Stabilized Iron Tailings Soil

The iron tailings soil was solidified with a cement dosage of 6% (by weight of the iron tailings soil). Water for mixing was substituted with varying concentrations of ISS solution (0.17%*m_cem_*, 0.33%*m_cem_*, and 1.67%*m_cem_*). Subsequently, Proctor compaction tests were conducted to determine the optimum moisture content and maximum dry density of specimens prepared with different ISS solution concentrations.

After determining the optimum moisture content and maximum dry density for specimens at various ISS concentrations, cylindrical compressive strength specimens with both a diameter and height of 50 mm were prepared according to JTG 3441-2024 [24] for testing their unconfined compressive strength. The concrete composition of cement-stabilized iron tailings soil with different ISS content is shown in Table 5.

#### 2.2.3. The Effect of ISS on Cement Hydration Under Pressure Compaction

The cement-stabilized iron tailings soil specimens were all prepared via pressure compaction, which constitutes a significant deviation from the conventional casting method employed for ordinary cement paste. To delve deeper into the influence of ISS on cement hydration during pressure compaction, the cement pastes with 1.67% ISS dosage (relative to the binder material) were employed both casting and pressure compaction (at 30 kN) methods for specimen fabrication. A consistent water-to-cement ratio of 0.26 was adopted to ensure the paste’s ability to be vibrated and consolidated after pouring, while also facilitating the molding process under pressure [25]. Subsequently, the hydration products of these specimens were analyzed at various curing times through XPS analysis.

### 2.3. Methods

In order to study the mechanism of ISS in the cement system and the cement-stabilized iron tailings soil system, the effects of ISS on the mechanical and microscopic properties of the two systems were tested, respectively.

#### 2.3.1. Test and Characterization Method of ISS in Cement System

##### Mechanical Properties

Cement mortar specimens with different contents of ISS were prepared using a water-to-cement ratio of 0.5, and the compressive and flexural strengths of different ages (7 d, 28 d, and 60 d) were measured. The specimens were cured under standard conditions according to GB/T 17671-2021 [26] with 20 ± 2 °C and ≥95% RH. According to the standard, the sample is a prismatic sample of 40 × 40 × 160 mm^3^. There are three groups of six samples in each group for testing compressive strength. There are three groups of samples used to test the flexural strength, with three samples in each group. The average value of the strength tests under different ages is taken as the final result. The purpose is to study the effect of ISS on strength development and to relate it to the hydration process.

The broken sample was immersed in isopropanol to suspend the hydration process as the recommended samples with the least damage to the hydration products [27].

##### Isothermal Calorimetry

The hydration heat of cement paste was measured by isothermal calorimeter at 20 ± 0.02 °C. The water-to-cement ratio was 0.5, and the dosages of ISS were 0.17%, 0.33%, and 1.67% by cement weight. The external stirring method was used.

Putting the ampoule into the calorimetric channel, the inevitable heat fluctuation will occur during the period of time when the measurement begins. To minimize the error, the integral of the accumulated heat was started from 0.75 h [28].

##### X-Ray Diffraction (XRD)

To examine the impact of ISS on phase composition evolution during cement hydration, specimens of varying hydration durations were analyzed using a D8 Advance X-ray diffractometer manufactured by Bruker Analytical Instruments, Karlsruhe, Germany. Utilizing Cu Kα radiation (λ = 0.154 nm). Samples were fractured at designated curing times and immersed in isopropanol, followed by vacuum drying at 40 °C for 48 h and pulverization to a particle size <20 µm. Based on the Rietveld method, different minerals in cement were quantitatively analyzed. The acquired data were processed with V6 version TOPAS software, employing corundum as an external standard, and phase quantification was achieved through Rietveld refinement calculations.

##### Scanning Electron Microscope (SEM)

The model GeminiSEM360 field emission scanning electron microscope produced by Carl Zeiss AG in Baden-Wurttemberg, Germany was used in this experiment. The sample used to observe the effect of ISS on the morphology of cement hydration products was taken from cement slurry. After reaching the prescribed curing age, the core was broken and cored. The core sample was soaked in isopropyl alcohol for 72 h to terminate hydration, and then dried in a vacuum drying oven at 40 °C. The sample used to observe the effect of ISS on the morphology of hydration products in cement-solidified iron tailings soil was taken from a cylindrical specimen core with a diameter and height of 50 mm. The remaining steps were the same as above. After the sample was dried, the samples were observed on a field emission scanning electron microscope.

##### Low-Field Nuclear Magnetic Resonance (NMR)

The low field nuclear magnetic resonance analyzer MacroMR12-110H-GS produced by Niumag Company in Suzhou, China was used in this experiment and it assessed porosity and pore distribution in cement-solidified soil, with/without ISS, under 0.3 T magnetic field, 25 mm coil, and 20 MHz frequency. The sample of the cement system is a 2 × 2 × 2 cm^3^ cube test block, and the sample of the cement-stabilized iron tailings soil system is a cylindrical test block with a diameter and height of 2 cm. Before the test, isopropyl alcohol was soaked for 72 h to terminate hydration and was dried at 40 °C for 48 h. High-pressure water saturation was then carried out to test the porosity of the sample.

##### X-Ray Photoelectron Spectroscopy (XPS)

The test samples were taken from the core of the sample in the respective system, and the powder was then tested. XPS was used to analyze the changes of hydration products of cement with ISS in different states. This was conducted utilizing the ESCALAB Xi+ X-Ray Photoelectron Spectroscopy, manufactured by Thermo Fisher Technologies in Massachusetts, USA. Which employed monochromatic Al Kα radiation (operating at 225 W, with a current of 15 mA and a voltage of 15 kV) in conjunction with low-energy electron flooding for effective charge neutralization and compensation. To mitigate the influence of surface charge effects, the characteristic C1s hydrocarbon peak located at a binding energy of 284.80 eV was employed as a reference for calibrating all subsequent binding energy measurements.

#### 2.3.2. Test and Characterization Method of ISS in Cement-Stabilized Iron Tailings System

##### Proctor Compaction Test

The Proctor compaction test was conducted in accordance with JTG 3441-2024 [24]. Approximately 400–500 g of the mixture was placed as the first layer into the compaction mold and compacted using a compaction apparatus with 27 blows (the compacted sample occupying approximately one-fifth of the total height of the test mold). After compaction, the surface was roughened using a soil scraper, and the second layer of mixture was added and compacted again. This compaction procedure was repeated five times in total. Upon completion of compaction, the top of the mold was leveled off using a straightedge, and the specimen was demolded, weighed, and the corresponding dry density and moisture content were calculated.

##### Unconfined Compression Strength (UCS)

The automatic road strength tester is used for testing to ensure that the pressure down speed of the indenter is 1.0 mm/min. The specimen is a cylindrical specimen with a diameter and height of 50 mm. On the last day of the specified curing period, the sample was saturated with water for 24 h, and then the surface water of the sample was wiped dry for testing.

##### Non-Evaporable Water Content

To elucidate the effect of the ISS environment on cement hydration in solidified iron tailings soil, a rigorous investigation of the non-evaporable water content (*W_ne_*) in various samples was undertaken. *W_ne_* was determined by the differential weight of specimens dried at 105 °C and 950 °C, accounting for contributions from iron tailings soil and cement [29]. The calculation employed the equation:

(1) $$W_{ne}=\frac{W_{1}-W_{2}}{W_{2}}-L_{c}-L_{i}$$

where W_1_ was the weight of the specimen after heating 105 °C (g), W_2_ was the weight of the specimen after heating 950 °C (g), L_c_ was the loss of ignition of fly ash (%), and L_i_ was the loss of ignition iron tailings soil (%).

##### Zeta Potential

To examine the transformation in surface charge subsequent to the adsorption of ISS onto the surface of iron tailings soil particles, a Zeta-sizer LAB instrument produced by Malvern Instrument Company in the Worcestershire, UK was employed to quantify the Zeta potential of the soil suspended in ISS solutions. The concentrations of the ISS solutions were precisely calibrated at 0%, 0.17%, 0.33%, and 1.67% (based on the mass of ISS utilized). Prior to the addition of the powdered iron tailings soil, the solutions were subjected to magnetic stirring for 5 min to ensure homogeneity. Subsequently, the liquid-solid mixture was agitated at a 50:3 ratio for 120 min at an ambient temperature of 25 °C, allowing for thorough interaction between the ISS and iron tailings soil particles. Following this equilibration period, the supernatant was isolated and subjected to Zeta potential measurements.

## 3. Results and Analysis

### 3.1. Mechanical Properties

#### 3.1.1. Effect of ISS on the Hydration Process and Mechanical Properties of Cement

##### Isothermal Calorimetry and Degree of Cement

**Figure 2:**
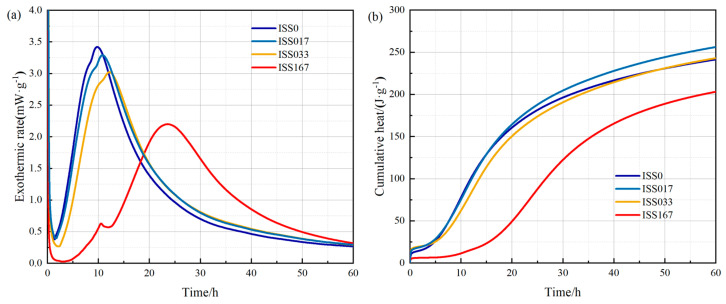
Thermal power and cumulative heat of hydration of cement with 0, 0.17%, 0.33%, and 1.67% ISS (**a**) the relationship between exothermic rate and time and (**b**) the relationship between cumulative heat and time.

The measured thermal power and cumulative heat curves of cement with different ISS contents are shown in Figure 2, and the cumulative hydration heat at different times is recorded in Table 5. The hydration heat release curve shows that ISS delays the hydration process of cement, and the effect is more obvious with the increase of ISS content. In addition, the maximum measured thermal power of the main hydration peak decreased with the increase of ISS content, and the shoulder of the pastes with ISS became more pronounced, indicating an increased second C_3_A reaction, which indicated the formation of second ettringite. However, the cement paste containing 1.67% ISS exhibited a broad exothermic peak, merging the peaks of C_3_A and C_3_S, and the hydration time was delayed by approximately 25 h.

As shown in Table 6, the cumulative heat release of ISS017 and ISS033 increased in the first hour compared with the blank sample. The reason may be that ISS caused the dissolution of the initial phase in the cement, including the gypsum phase, which promoted the formation of the initial ettringite phase. However, when the ISS content was 1.67%, the cumulative hydration heat decreased rapidly at 1 h.

After 44 h, the cumulative heat measured by 0.17% ISS was slightly higher than that of the reference paste, 0.33% ISS was equivalent to the reference sample, and 1.67% ISS cumulative heat was much lower than the reference.

##### The Effect of ISS on Mechanical Properties

**Figure 3:**
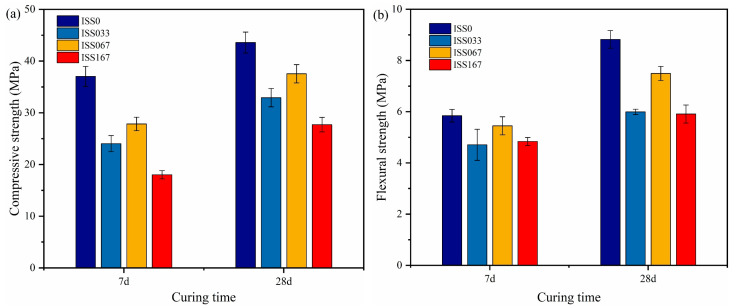
The compressive and flexural strength of ISS cement mortar with different content of ISS cement mortar (**a**) relationship between compressive strength and curing age and (**b**) relationship between flexural strength and curing age.

Figure 3 shows the effect of ISS on the mechanical properties’ development of mortar. The compressive and flexural strength of cement mortar was adversely affected by ISS at any curing time. However, the strength of cement mortar increased with the increase of ISS content. The compressive strength of cement mortar at 7 d decreased by 59.8%, 44.1%, and 29.1%, respectively, while the ISS content was 1.67%, 0.33%, and 0.17%, respectively. During the 7 to 28 d curing time, the compressive strength of the samples with ISS exhibited a linear increase, while the strength of ISS0 increased at a slower rate after 28 d. At 7 d, the flexural strength of the specimens with ISS decreased by approximately 17% compared to the blank sample. This suggests that the varying content of ISS had minimal impact on the flexural strength at an early age. However, the flexural strength of the specimens with ISS increased slowly at 28 d.

#### 3.1.2. Effect of ISS on Mechanical Properties of Cement-Stabilized Iron Tailings Soil

##### Compaction Characteristic

**Figure 4:**
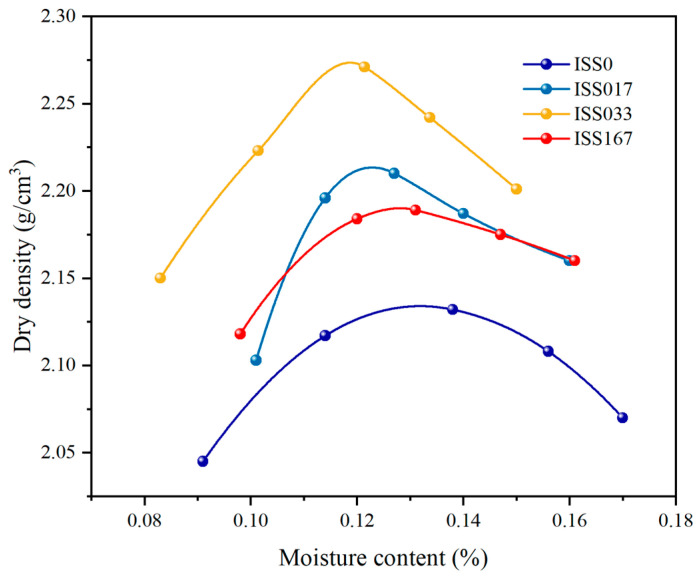
Compaction curve of cement-stabilized iron tailings soil with different ISS content.

Figure 4 depicts the compaction behavior of cement-stabilized iron tailings soil as a function of varying ISS content. Upon the addition of ISS, a notable increase in the maximum dry density of the samples is observed, accompanied by a corresponding decrease in the optimum moisture content. At an ISS content of 0.33%, the dry density of the specimens attains its maximum value, surpassing that of the control sample by a significant 6.5%. Conversely, when the ISS content is escalated to 1.67%, a discernible downward trend in the dry density is evident, suggesting that an excessive ISS content does not confer any further enhancement in engineering properties. This finding underscores the existence of an optimal ISS dosage within the cement-stabilized iron tailings soil matrix.

##### UCS

**Figure 5:**
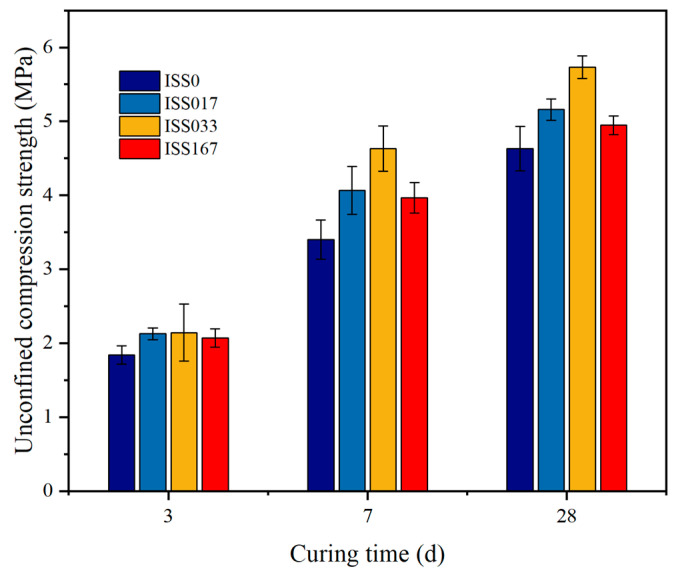
UCS of specimens at different curing times.

Figure 5 presents the UCS of cement-stabilized iron tailings soil incorporating varying ISS contents. The incorporation of ISS serves to augment the strength characteristics of the cement-stabilized iron tailings soil matrix. When compared to the control specimen, a notable enhancement in UCS is observed, with a 35.3% and 24.1% increase at 7 d and 28 d of curing, respectively, at an ISS content of 0.33%. Furthermore, even at other ISS concentrations, the UCS exhibits a pronounced improvement over the control sample. This phenomenon can be attributed to the ISS’s capability to elevate the maximum dry density of the cement-stabilized iron tailings soil. The consequential rise in dry density, indicative of an increase in solid-phase compactness and a corresponding decrease in porosity, thereby underpins the enhancement in mechanical properties.

### 3.2. The Pore Distribution of the Hardened Cement Paste

**Figure 6:**
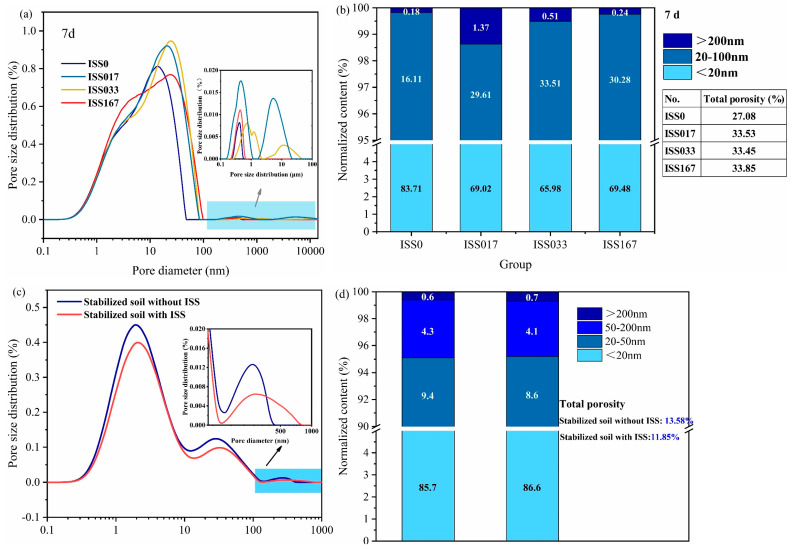
The (**a**) pore distribution and (**b**) total porosity of hardened cement paste; (**c**) pore distribution and (**d**) total porosity of cement-stabilized iron tailings soil.

Figure 6a,b illustrates the pore size distribution and total porosity, respectively, of distinct cement pastes at 7 d. The total porosity of samples incorporated with ISS was consistently higher than that of the control samples. The majority of pores in all specimens were distributed within the 1–100 nm range. With extended curing periods, the pore size distribution of the control samples shifted more towards the 1–20 nm range, whereas the pore size distribution of the ISS-doped samples did not exhibit significant changes. Notably, at an ISS dosage of 0.33%, the total porosity of the specimens was the lowest, and the proportion of detrimental pores was minimized, which correlates with the compressive strength findings. Overall, the incorporation of ISS did not refine the pore structure of the cement paste but rather increased the porosity of the specimens, thereby contributing to the decrease in the strength of the cement paste.

However, in cement-stabilized iron tailings soil, the samples incorporated with ISS exhibited significantly lower porosity compared to the control samples (Figure 6c,d). Specifically, at an ISS dosage of 0.33%, the porosity was reduced to 11.85%, marking a 13% decrease from the control. This reduced porosity can be attributed to the achievement of higher density and lower water demand in the ISS-modified samples under similar compaction efforts, indicative of a more compact solidified soil structure. Consequently, the incorporation of ISS contributed to the observed lower porosity, which was one of the mechanisms underlying its ability to enhance the performance of cement-stabilized iron tailings soil.

It was evident that ISS exerted an adverse effect on the mechanical properties of cement; paradoxically, it contributed to enhanced strength in cement-stabilized iron tailings soil, partially attributed to the increase in dry density, which was related a reduction in porosity. This raises another question of whether ISS influences cement hydration in the context of cement-stabilized iron tailings systems, and what differences exist in the resulting hydration products. To address this, an analysis of the type and morphology of hydration products is essential.

### 3.3. Analysis of Hydration Products

#### 3.3.1. Mineralogical Analysis

**Figure 7:**
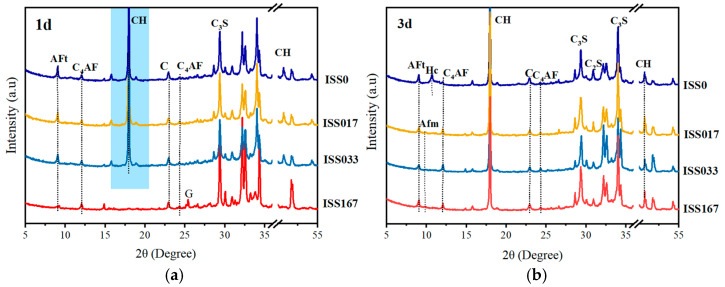
XRD of cement pastes with different ISS content and curing times (**a**) hydration time is 1 d and (**b**) hydration time is 3 d.

Figure 7 presents the XRD patterns of cement paste incorporating varying concentrations of ISS at distinct curing ages. The incorporation of ISS did not elicit a change in the types of hydration products formed. However, it was observed to delay the emergence of diffraction peaks corresponding to these hydration products. Specifically, at a curing age of 1 day, the sample containing 1.67% ISS exhibited a notably weakened diffraction peak for AFt (Ettringite), with no discernible peak for CH (Ca(OH)_2_) being detected. Furthermore, the presence of gypsum diffraction peaks suggested that excessive ISS concentrations hindered the dissolution of the gypsum phase. Conversely, at 3 d of curing, samples with ISS concentrations of 0.17% and 0.33% exhibited XRD patterns comparable to those of the control samples, indicating similar phase compositions.

**Figure 8:**
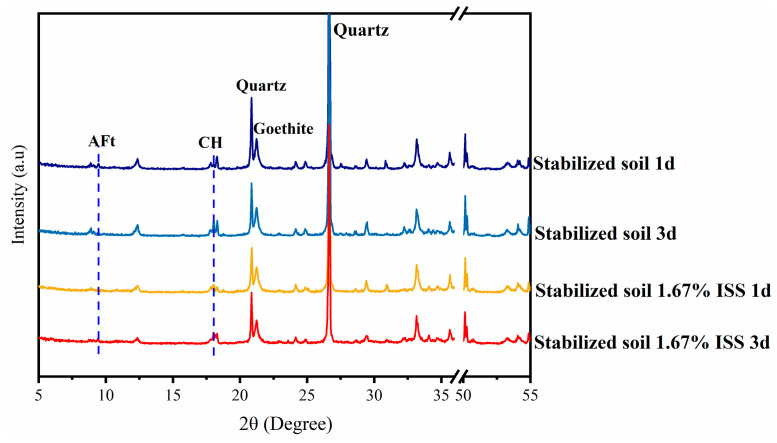
XRD of cement iron stabilized tailings samples with and without ISS.

However, the XRD patterns of cement-stabilized iron tailings exhibited peaks attributed to ettringite and calcium hydroxide CH at all ages examined (Figure 8), which was notably distinct from the XRD results of cement paste at 1 day. Specifically, under a 1.67% incorporation of ISS, the diffraction peak of CH was barely discernible in the XRD of cement at 1-day age, whereas it was relatively prominent in the XRD of cement-stabilized iron tailings. This observation indicated a mitigated retardation effect of ISS on the early hydration of cement in cement-stabilized soil.

#### 3.3.2. Morphology Analysis

**Figure 9:**
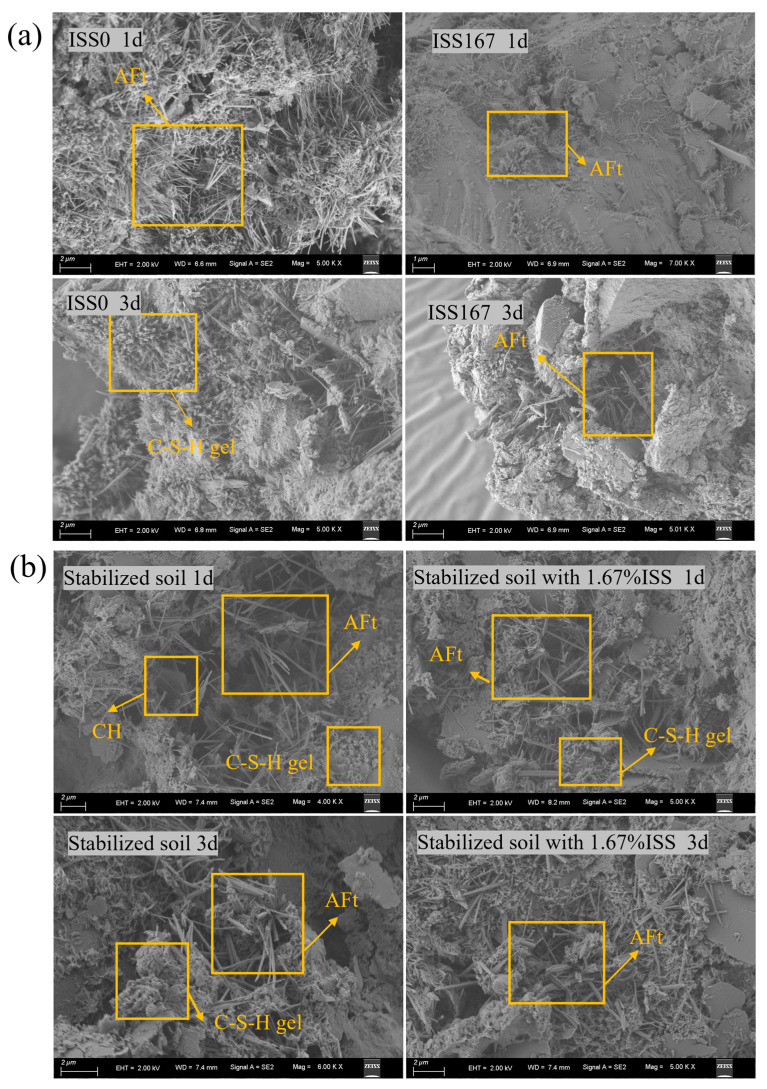
(**a**) SEM images of cement paste with and without ISS; (**b**) SEM images of cement-stabilized iron tailings soil with and without ISS.

Figure 9 exhibits 1 d and 3 d SEM micrographs of specimens with varying ISS concentrations. Conspicuous needle-shaped AFt, hexagonal CH platelets, and C-S-H gel are discernible in all samples. At 1 d, the control specimen exhibits extensive AFt formation, enshrouding C-S-H gel and cement particles, whereas AFt precipitation in ISS-incorporated samples is markedly reduced, especially at 1.67% ISS concentration, revealing minimal AFt. Over 3 d, hydration product abundance gradually augments in ISS-containing samples.

However, at both 1 d and 3 d curing times, AFt was observed to intertwine around the iron tailings soil particles in all samples of cement-stabilized iron tailings soil (Figure 9b). In contrast to the cement paste with a dosage of 1.67%, the presence of distinct AFt in the cement-stabilized iron tailings soil at 1 d curing age with an ISS dosage of 1.67% indicated a mitigated early-stage inhibition effect of ISS on hydration products within the iron tailings soil.

ISS exhibits distinct roles in cement paste and cement-stabilized iron tailings soil systems. Consequently, we employed the Rietveld method and non-evaporable water content analysis separately to quantitatively assess the hydration product content in cement paste and cement-stabilized iron tailings soil, respectively.

### 3.4. Quantitative Analysis of Hydration Products

#### 3.4.1. Quantitative Analysis Using the Rietveld Method

**Figure 10:**
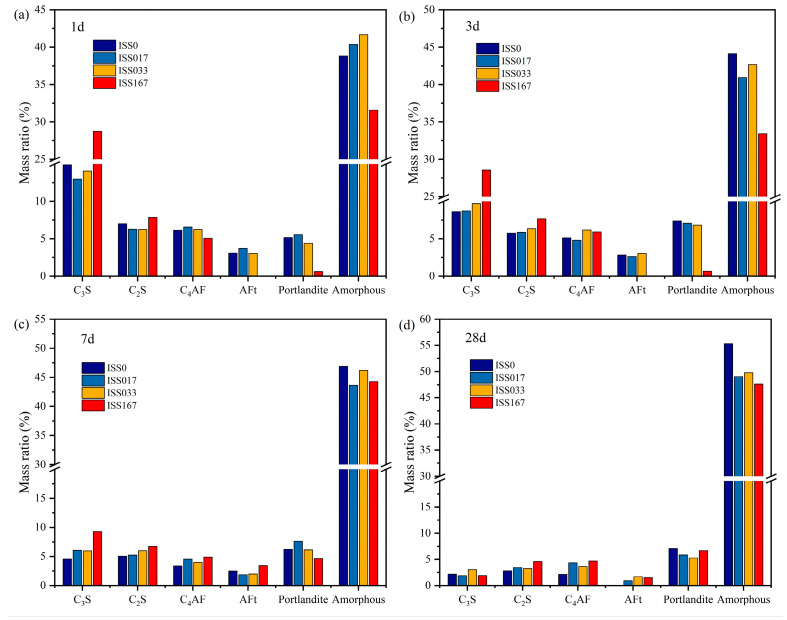
Cement minerals and hydration products in different periods quantified by the Rietveld method (**a**) hydration time is 1 d and (**b**) hydration time is 3 d; (**c**) hydration time is 7 d and (**d**) hydration time is 28 d.

Figure 10 presents a quantitative analysis of the degree of mineral hydration and hydration products, as determined through Rietveld refinement, across varying concentrations of ISS. Notably, the consumption rates of C_3_S, C_2_S, and C_4_AF in samples containing ISS were significantly reduced compared to the control sample, indicating that the addition of ISS inhibits the hydration reaction. This inhibitory effect was further corroborated by the observed trends in hydration product formation. Specifically, at 1 day of hydration, the sample containing 1.67% ISS exhibited a negligible quantity of AFt and CH, suggesting a substantial delay in the hydration process. Conversely, the sample with 0.33% ISS demonstrated the highest degree of hydration among all ISS-containing samples, with this trend persisting until the age of 28 d. It is worth noting that in the samples added with ISS, when the ISS content was 0.33%, the hydration products of the specimen were the most, especially the C-S-H gel, which was also reflected in the strength.

#### 3.4.2. Non-Evaporable Water Content in Cement-Stabilized Iron Tailings Soil

**Figure 11:**
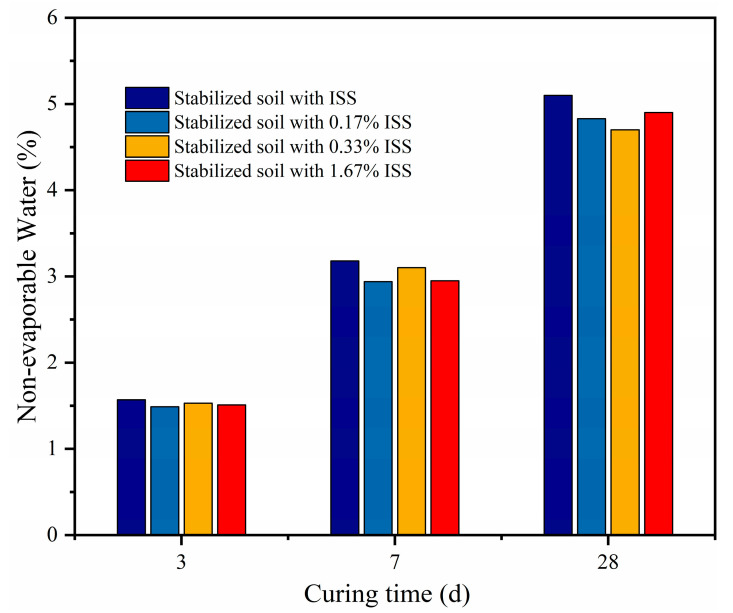
Non-evaporable water content of samples.

To gain insights into the extent of cement hydration within cement-stabilized iron tailings soil, we conducted an analysis of the non-evaporable water content of these materials. Figure 11 illustrates the variation in non-evaporable water content within cement-stabilized iron tailings soil at 7 d and 28 d curing intervals, contingent upon varying dosages of ISS. A discernible trend emerged, indicating a decline in the non-evaporable water content subsequent to the incorporation of ISS. Specifically, at a 3 d reference point, comparative to the control specimen, the samples with ISS dosages of 0.17%, 0.33%, and 1.67% exhibited respective reductions of 5.1%, 2.5%, and 3.8% in their non-evaporable water content.

The introduction of ISS into cement-stabilized iron tailings soil was observed to attenuate the hydration kinetics of cement, resulting in a weakened hydration process. Notably, even at the highest dosage of 1.67% within the cementitious matrix, ISS failed to contribute appreciably to the overall strength development, highlighting its inability to promote cementitious reactions. Given that the cement-stabilized iron tailings soil was manufactured through a compaction process, characterized by a relatively low water-to-cement ratio, the retarding influence imparted by the admixture on cement hydration became less pronounced.

### 3.5. The Role of ISS in Cement-Stabilized Iron Tailings Soil

In the cement hydration system, ISS apparently exerts a detrimental effect, leading to a decrease in the mechanical properties of cement and a reduction in the quantity of hydration products. However, in cement-stabilized iron tailings soil, ISS paradoxically enhances its strength. This can be attributed to two aspects: Firstly, ISS increases the dry density of cement-stabilized iron tailings, resulting in improved compactness; secondly, the delaying effect of ISS on cement hydration becomes less pronounced in cement-stabilized iron tailings soil, which may be associated with pressure compaction.

In this section, Zeta potential measurements are utilized to examine the variations in surface charge of iron tailings soil particles, thereby elucidating the influence of ISS on dry density. Moreover, cement specimens were compacted and subjected to XPS analysis to gain a deeper understanding of the attenuation of the delaying effect of ISS on cement hydration under different compaction methods.

#### 3.5.1. Zeta Potential

The implementation of ISS enhances the compaction profile of cement-stabilized iron tailings soil, enabling the specimens to attain a heightened dry density under identical compaction energy inputs. To elucidate the underlying mechanisms responsible for this altered compaction behavior, we conducted a systematic investigation of the Zeta potential on the surface of iron tailings soil particles across varying ISS concentrations. This analysis aimed to characterize the electrostatic repulsion forces operating between the iron tailings soil particles, thereby providing a rationale for the observed modifications in the compaction characteristics [30].

**Figure 12:**
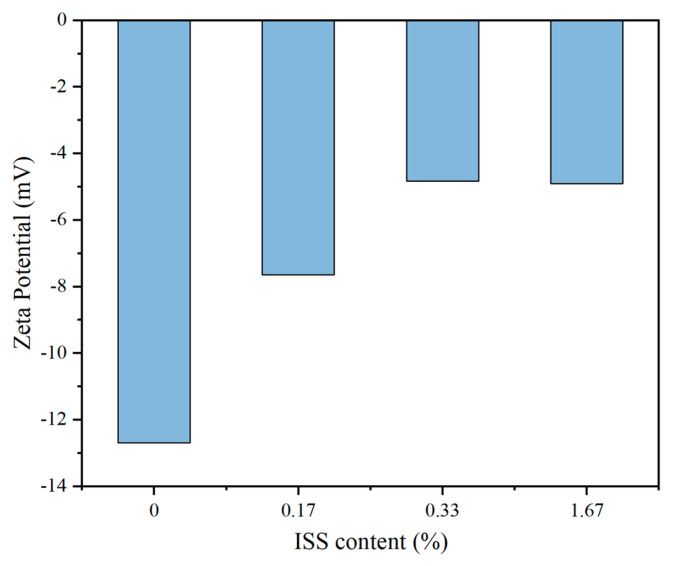
Zeta potential of iron tailings soil particle surface with different ISS content.

Figure 12 depicts the variation in Zeta potential on the surface of iron tailings soil particles as a function of different ISS dosages. Notably, the application of ISS significantly diminishes the Zeta potential on the surface of iron tailings soil particles. Specifically, at an ISS content of 0.33%, a substantial decrease of 62% in Zeta potential is observed relative to the control sample. However, further increments in ISS content yield diminishing returns in terms of reducing the Zeta potential, suggesting that an optimal adsorption capacity of ISS on the tailings particles had been achieved. This reduction in surface electronegativity of iron tailings soil particles, mediated by ISS, translates into a decrease in repulsive forces between particle surfaces [31]. Consequently, under identical pressure conditions, the particles exhibit a greater propensity for flocculation, ultimately leading to an enhancement in the dry density of the soil.

#### 3.5.2. Changes of Hydration Products Under Compaction Conditions

**Figure 13:**
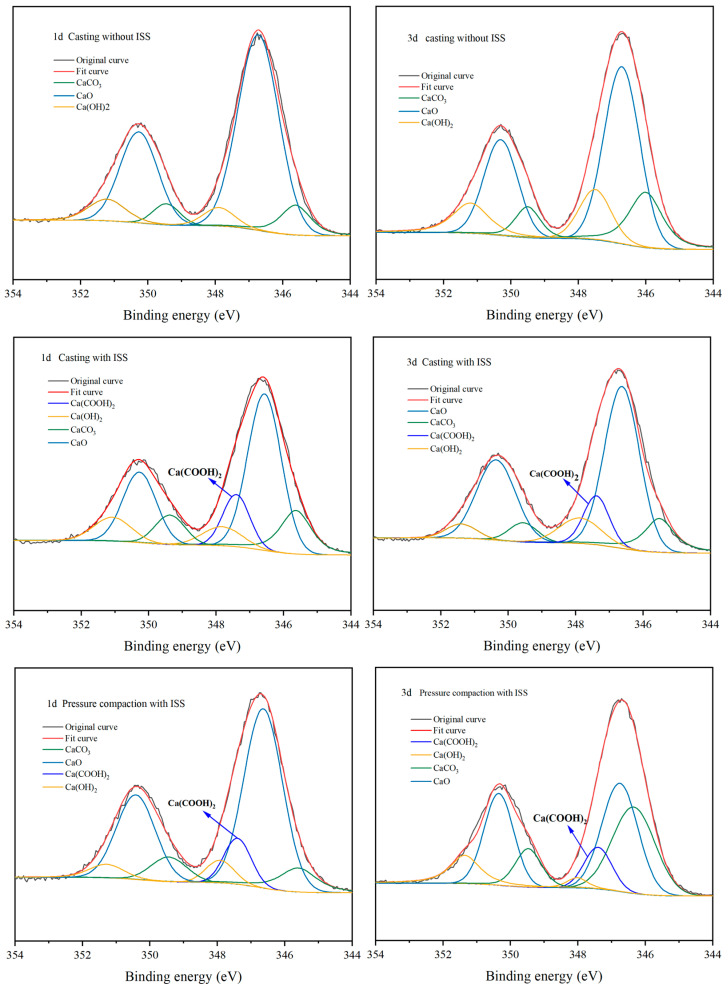
Ca2p peak fitting for different molding methods of cement.

Figure 13 presents the XPS spectra of cement samples prepared via casting and pressure-compaction methods. The accuracy of the fitting results was verified by assessing the degree of congruence between the fitted curves and the original spectra. Calcium (Ca) in all samples was identified to exist in three distinct chemical environments, namely, CaO, Ca-OH, and CaCO_3_, with peak separation and fitting performed for Ca in these varying chemical states [25]. Notably, in samples incorporating industrial starch sludge (ISS), a novel compound, Ca(COOH)_2_, emerged at 347.4 eV in the spectra, aligning with previous findings [32,33]. This observation reinforces the notion that ISS can form complexes with Ca^2+^ ions in cement, adsorbing onto mineral surfaces and thereby influencing the hydration degree.

**Figure 14:**
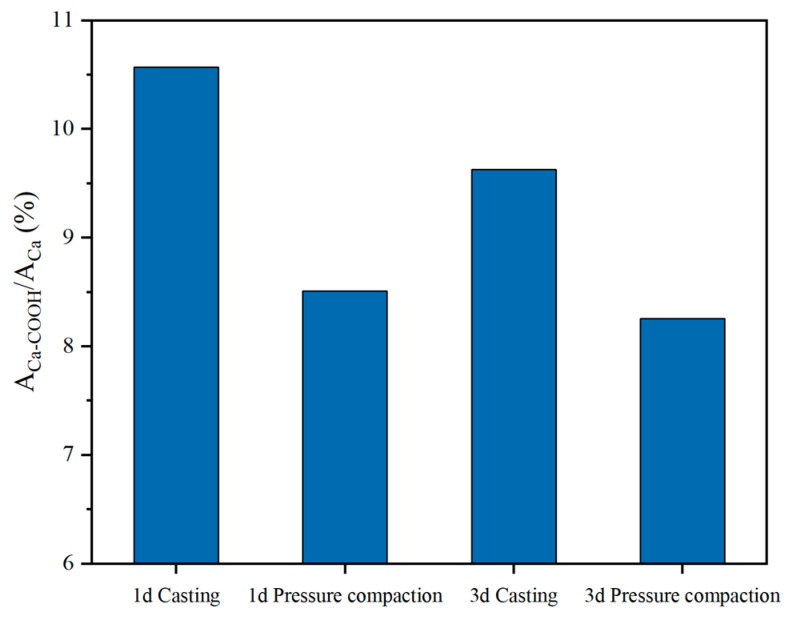
Contents of new phase Ca(COOH)_2_ in the samples.

Figure 14 illustrates the proportion of Ca(COOH)_2_ relative to total calcium under different forming techniques. For cast slurries, the proportions at 1 d and 3 d were 10.6% and 9.6%, respectively, whereas for pressure-compacted samples, these values decreased significantly to 8.5% and 8.2%, respectively. The reduction in Ca(COOH)_2_ quantity suggests a diminished presence of complexes adsorbed on cement mineral surfaces, subsequently mitigating their impact on cement hydration. Furthermore, within the composite system, cement particles must overcome energy barriers to facilitate hydration reactions. Pressure-compaction enhances interparticle contact area, lowering the energy barrier height and accelerating the reaction rate.

### 3.6. Discussion

#### 3.6.1. The Effect of ISS on the Hydration Properties of Cement

The incorporation of industrial by-product ISS was found to retard the comprehensive progression of cement hydration dynamics, a phenomenon attributed to the presence of carboxyl and hydroxyl functional groups inherent in ISS [34,35]. Specifically, at an ISS concentration of 1.67%, exceeding the conventional range for cement admixtures, the specimens exhibited pronounced retardation in hydration kinetics. Consequently, this elevated dosage led to a notable decrease in the initial heat evolution rate, with the cumulative heat release measured at 1 d hydration time amounting to merely 80.2 J, representing a 54.7% diminution compared to the reference specimen without ISS. Further analysis through XRD revealed the persistence of discernible gypsum phases, suggesting that the hydration process was impeded. This retardation can be rationalized by the excessive adsorption of ISS molecules onto the surface of cement particles, facilitating the formation of an ISS film that obstructs the interfacial contact between water and cement particles, thereby delaying the dissolution of cementitious components. Consequently, at 1 d of hydration, the cement paste containing 1.67% ISS exhibited predominantly weak AFt formation, indicative of a retarded hydration pathway.

At ISS concentrations of 0.17% and 0.33%, the carboxyl group undergoes chemical adsorption onto the active sites of cement particles [36]. This process, facilitated by robust coordination interactions, enables preferential binding of calcium ions present in cement clinker minerals [37]. Consequently, at these ISS concentrations, the cumulative heat release of cement within 24 h surpasses that of the control specimen, corroborated by un-hydrated mineral quantification via Rietveld analysis. Specifically, the consumption of C_3_S and C_2_S at 24 h is enhanced compared to the control. However, the complexation between ISS and calcium ions, along with potential aluminum ions, forms a covering layer on the cement particle surfaces, which hinders further hydration progression. This phenomenon translates into reduced hydration product formation in cement pastes blended with ISS at 3 d, 7 d, and 28 d, when compared to the control. Furthermore, the porosity of these samples at 7 d and 28 d is notably higher, manifesting macroscopically as a decrease in compressive strength.

The overarching influence of ISS on cement hydration can be concisely encapsulated as follows: In the initial hydration phase, the rapid dissolution of clinker phases, notably gypsum, C_3_A, and C_3_S, is expedited by the complexation of ISS with calcium and aluminum ions present in the cement matrix. Consequently, there is an elevated consumption of C_3_A and C_3_S during this initial period. However, the subsequent formation of Ca(COOH)_2_ complexes on the C_3_S surface acts as a hindrance, manifesting as a pronounced inhibitory effect on C_3_S hydration within the 3 to 7 d time frame. As cement hydration progresses at a more leisurely pace, the proliferation of hydration products and the consequential expansion of surface area facilitate the desorption of Ca(COOH)_2_ complexes previously adsorbed around C_3_S. Additionally, these hydration products actively consume residual ISS, thereby catalyzing the acceleration of C_3_S and C_2_S hydration rates.

#### 3.6.2. The Effect of ISS on the Compaction Performance of Cement-Stabilized Soil

Despite its detrimental effects on cement hydration and strength development, ISS exhibits a beneficial role in the context of cement-stabilized iron tailings soil. Specifically, the incorporation of ISS enhances the dry density of the specimen, resulting in an increase in the consolidated bulk density of the stabilized matrix. This, in turn, contributes to a notable improvement in the UCS of the cement-stabilized iron tailings soil.

The surface of iron tailings soil particles is primarily characterized by a double-layer structure dominated by monovalent ions, whereas the introduction of ISS can disrupt this double-layer structure, resulting in a decrease in its thickness [31,38], thereby altering the interparticle interactions. Consequently, the collapse of the electric double-layer structure facilitates the liberation of interlayer water, enabling the achievement of MDD (Maximum dry density) with reduced water content. Compaction experiments confirm a decrease in the OMC (Optimum moisture content) under these conditions.

However, an interesting trend emerges when the ISS content surpasses 0.33%. The dry density of the cement-stabilized iron tailings soil declines, indicating that an excess of ISS on the iron tailings soil surface augments intermolecular repulsive forces. When these repulsive forces surpass the flocculating effect imparted by ISS, the degree of compaction diminishes under a constant compaction effort, as documented in previous works [16,39]. This phenomenon accounts for the observed increase in OMC and the corresponding decrease in MDD beyond 0.33% ISS content. Nevertheless, the overall application of ISS in iron tailings soil base enhancement is advantageous, as it consistently enables the attainment of higher dry densities and lower water contents under equivalent compaction energies. This enhancement is pivotal in optimizing the physical performance indicators of the stabilized soil, thereby underscoring the viability of ISS as a stabilizer in this context.

#### 3.6.3. The Effect of ISS on the Degree of Cement Hydration in Cement-Stabilized Soil

The incorporation of ISS into cement paste has been observed to retard the hydration kinetics of clinker, particularly during the initial stages, resulting in a diminished strength development at an early age (e.g., 3 d) for cement paste containing 1.67% ISS. However, this adverse effect is mitigated in the context of cement-stabilized iron tailings soil. During the assessment of non-evaporable water content, while ISS continues to exert an influence on cement hydration, its impact becomes negligible. This attenuation can be attributed to the unique compaction characteristics of cement-stabilized iron tailings soil, which facilitates the attainment of a dense microstructure that mitigates the disruptive effects of ISS on hydration processes.

The presence of ISS exerts a hindering effect on the hydration of cement, whereby the formation of a complex between ISS and calcium ions encapsulates the surface of C_3_S. However, the presence of external pressure alters the amount of complex formation. During the pouring and vibration molding process, the quantity of complexes formed between ISS and Ca^2+^ is significantly higher than that in specimens subjected to compaction molding. This observation implies that in cement-stabilized iron tailings soil, there are fewer complexes covering the surface of cement minerals, thereby reducing their inhibitory effect on cement hydration. Additionally, in the context of compaction molding, the application of external pressure augments the interfacial area between cement particles, thereby reducing the height of the energy barrier and enhancing the reaction rate constant. Consequently, this promotes the hydration of cement, demonstrating that despite ISS’s pronounced influence in cement paste, its effects become negligible in cement-stabilized iron tailings soil [25,40].

## 4. Conclusions

This study investigates the influence of ISS on cement hydration and analyzes its reinforcing effect within cement-stabilized iron tailings soil. The specimens of cement paste and cement-stabilized iron tailings soil were prepared with different contents of ISS. The effects of pores and hydration products on mechanical properties were analyzed, and the mechanism of ISS in cement paste, cement mortar, and cement-stabilized iron tailings soil were also discussed. The results of the subsequent research are summarized as follows:

(1) ISS weakens the mechanical properties of cement. When ISS content is 1.67%, the 7 d compressive strength of cement mortar decreases by 59.8% compared with the reference group. This retardation arises due to carboxyl and hydroxyl moieties in ISS forming complexes with Ca^2+^, creating a barrier on cement particle surfaces.

(2) ISS reduces the Zeta potential by thinning the double electric layer structure on the surface of iron tailings particles, thereby improving the matrix compactness under the same compaction work and optimizing the mechanical properties of cement-stabilized iron tailings soil. When the ISS content is 0.33%, the 7 d unconfined compressive strength is increased by 35.3% compared with the reference group.

(3) Under the application of pressure, the retardation effect of ISS on cement hydration diminishes. This can be attributed to the reduced reaction between ISS and Ca^2+^ during the pressing and molding process, leading to a decrease in the number of complexes coating cement minerals, which subsequently mitigates the inhibitory effect of ISS on cement hydration.

## Figures and Tables

**Table 1:** Physical properties of cement.

Setting Time (min)		Compressive Strength (MPa)		Flexural Strength (MPa)		Fineness
Initial set	Final set	3 d	28 d	3 d	28 d	1.2
156	282	22.1	44.7	5.2	9.6	

**Table 2:** Chemical composition of cement.

Chemical Composition	CaO	SiO_2_	Al_2_O_3_	Fe_2_O_3_	MgO	SO_3_
Content (%)	58.67	24.16	6.42	4.03	0.83	2.21

**Table 3:** Chemical composition of iron tailings soil.

Component	Iron Tailings Soil (%)
SiO_2_	51.4
Al_2_O_3_	13.6
Fe_2_O_3_	10.2
CaO	6.58
MgO	5.07
SO_3_	3.84
K_2_O	3.06
Na_2_O	2.21
P_2_O_5_	1.03
TiO_2_	0.83
MnO	0.17
Loss	2.64

**Table 4:** Mix ratio of ISS and cement.

	Cement	Sand	W/C	ISS (%)
ISS0	450	1350	0.5	0
ISS017	450	1350	0.5	0.17%*m_cem_*
ISS033	450	1350	0.5	0.33%*m_cem_*
ISS167	450	1350	0.5	1.67%*m_cem_*

**Table 5:** The composition of different cement-stabilized iron tailings soil.

	Maximum Dry Density (g/cm^3^)	Optimum Moisture Content (%)	Sample Volume (cm^3^)	ISS (%)
ISS0	2.132	13.8	98.125	0
ISS017	2.210	12.7	98.125	0.17%*m_cem_*
ISS033	2.271	12.1	98.125	0.33%*m_cem_*
ISS167	2.189	13.1	98.125	1.67%*m_cem_*

**Table 6:** Cumulative heat of cement pastes with and without ISS (%).

Samples	Cumulative Heat Cement with Different ISS Content (J/g)				
	1 h	10 h	24 h	45 h	60 h
ISS0	13.3	77.4	177.7	224.3	241.3
ISS017	17.3	75.4	183.9	236.7	256.1
ISS033	18.6	62.5	169.8	223.4	243.0
ISS167	5.9	11.6	80.2	178.8	203.1

## Data Availability

The original contributions presented in the study are included in the article, further inquiries can be directed to the corresponding author.

## References

[1] Wang A., Zhang D., Deng Y. (2018). Lateral response of single piles in cement-improved soil: Numerical and theoretical investigation. Comput. Geotech..

[2] Qiang Y., Chen Y. (2015). Experimental Research on the Mechanical Behavior of Lime-Treated Soil under Different Loading Rates. Adv. Mater. Sci. Eng..

[3] Jallu M., Arulrajah A., Saride S., Evans R. (2020). Flexural fatigue behavior of fly ash geopolymer stabilized-geogrid reinforced RAP bases. Constr. Build. Mater..

[4] Little D.N., Nair S., Herbert B. (2010). Addressing Sulfate-Induced Heave in Lime Treated Soils. J. Geotech. Geoenviron. Eng..

[5] Intaj F., Liu Y., Wu Z. (2019). Application and Evaluation of Micro-Cracking on Cement-Stabilized Bases at Field Projects in Louisiana. Transp. Res. Rec..

[6] Al-Dakheeli H., Arefin S., Bulut R., Little D. (2021). Effectiveness of ionic stabilization in the mitigation of soil volume change behavior. Transp. Geotech..

[7] Katz L.E., Rauch A.F., Liljestrand H.M., Harmon J.S., Shaw K.S., Albers H. (2001). Mechanisms of Soil Stabilization with Liquid Ionic Stabilizer. Transp. Res. Rec..

[8] Arefin S., Al-Dakheeli H., Bulut R. (2021). Stabilization of expansive soils using ionic stabilizer. Bull. Eng. Geol. Environ..

[9] Gautam S., Hoyos L.R., He S., Prabakar S., Yu X. (2020). Chemical Treatment of a Highly Expansive Clay Using a Liquid Ionic Soil Stabilizer. Geotech. Geol. Eng..

[10] Huo J., Wang Z., Zhang T., He R., Chen H. (2021). Influences of interaction between cement and ionic paraffin emulsion on cement hydration. Constr. Build. Mater..

[11] Khan R.A., Murtaza M., Mustafa A., Abdulraheem A., Mahmoud M., Kamal M.S. (2023). Ionic liquids as clay stabilizer additive in fracturing fluid. Fuel.

[12] Kaur G., Kumar H., Singla M. (2022). Diverse applications of ionic liquids: A comprehensive review. J. Mol. Liq..

[13] Petry T.M., Das B. (2001). Evaluation of Chemical Modifiers and Stabilizers for Chemically Active Soils—Clays. Transp. Res. Rec..

[14] Eren Ş., Filiz M. (2009). Comparing the conventional soil stabilization methods to the consolid system used as an alternative admixture matter in Isparta Darıdere material. Constr. Build. Mater..

[15] Soltani A., Deng A., Taheri A., Mirzababaei M. (2017). A sulphonated oil for stabilisation of expansive soils. Int. J. Pavement Eng..

[16] Jia J., Lu X., Zhu J., Wang J., Zhang L., Cheng X. (2024). Effect of ionic soil stabilizer on the properties of lime and fly ash stabilized iron tailings as pavement base. Constr. Build. Mater..

[17] Zhang B., Jiang W., Xu Q., Yuan D., Shan J., Lu R. (2022). Experimental feasibility study of ethylene-vinyl acetate copolymer (EVA) as cement-stabilized soil curing agent. Road Mater. Pavement Des..

[18] Xue C., Wang X., Lian B., Luo L., Liu K. (2023). Research on the mechanism of composite improvement of loess based on quantitative analysis of microstructure and mechanical strength. Constr. Build. Mater..

[19] Latifi N., Eisazadeh A., Marto A. (2014). Strength behavior and microstructural characteristics of tropical laterite soil treated with sodium silicate-based liquid stabilizer. Environ. Earth Sci..

[20] Ma F., Wu B., Zhang Q., Cui D., Liu Q., Peng C., Li F., Gu Q. (2018). An innovative method for the solidification/stabilization of PAHs-contaminated soil using sulfonated oil. J. Hazard. Mater..

[21] Wu X.-T., Qi Y., Chen B. (2021). Solidification effect and mechanism of ionic soil stabilizer applied on high-water-content clay. B. Eng. Geol. Env..

[22] Ministry of Transport of the People’s Republic of China (2020). Test Methods of Soils for Highway Engineering.

[23] (2022). Technical Standard for Geotechnical Engineering of Tailings Embankmen.

[24] Ministry of Transport of the People’s Republic of China (2024). Test Methods of Materials Stabilized with Inorganic Binders for Highway Engineering.

[25] Wang B., Xing X., Lin F., Wang S., Li F., Huang H., Wei J., Yu Q. (2024). Effect of vacuum–vibration–compaction molding on the properties and performances of polymer-modified cementitious material. Cem. Concr. Compos..

[26] (2021). Test Method of Cement Mortar Strength (ISO Method).

[27] Zhang J., Scherer G.W. (2011). Comparison of methods for arresting hydration of cement. Cem. Concr. Res..

[28] Yan Y., Ouzia A., Yu C., Liu J., Scrivener K.L. (2020). Effect of a novel starch-based temperature rise inhibitor on cement hydration and microstructure development. Cem. Concr. Res..

[29] Ma Y., Ye G. (2015). The shrinkage of alkali activated fly ash. Cem. Concr. Res..

[30] Marchuk A., Rengasamy P., McNeill A. (2013). Influence of organic matter, clay mineralogy, and pH on the effects of CROSS on soil structure is related to the zeta potential of the dispersed clay. Soil Res..

[31] Farahani E., Emami H., Fotovat A., Khorassani R. (2019). Effect of different K:Na ratios in soil on dispersive charge, cation exchange and zeta potential. Eur. J. Soil Sci..

[32] Chen B., Wu K., Yao W. (2004). Conductivity of carbon fiber reinforced cement-based composites. Cem. Concr. Compos..

[33] Wang M., Wang R., Yao H., Farhan S., Zheng S., Wang Z., Du C., Jiang H. (2016). Research on the mechanism of polymer latex modified cement. Constr. Build. Mater..

[34] Chaudhari O., Biernacki J.J., Northrup S. (2017). Effect of carboxylic and hydroxycarboxylic acids on cement hydration: Experimental and molecular modeling study. J. Mater. Sci..

[35] Marchon D., Flatt R.J., Aïtcin P.-C., Flatt R.J. (2016). 12—Impact of chemical admixtures on cement hydration. Science and Technology of Concrete Admixtures.

[36] Danner T., Justnes H., Geiker M., Lauten R.A. (2015). Phase changes during the early hydration of Portland cement with Ca-lignosulfonates. Cem. Concr. Res..

[37] Jolicoeur C., Simard M.-A. (1998). Chemical admixture-cement interactions: Phenomenology and physico-chemical concepts. Cem. Concr. Compos..

[38] Kiventerä J., Piekkari K., Isteri V., Ohenoja K., Tanskanen P., Illikainen M. (2019). Solidification/stabilization of gold mine tailings using calcium sulfoaluminate-belite cement. J. Clean. Prod..

[39] Onyejekwe S., Ghataora G.S. (2015). Soil stabilization using proprietary liquid chemical stabilizers: Sulphonated oil and a polymer. Bull. Eng. Geol. Environ..

[40] Zhu G., Luo Y., Chen C., Shen K., Zhang Y. (2023). Effect of metakaolin on the microstructure of cement-based artificial marble slabs incorporating natural marble offcuts under vacuum vibration compaction method. Constr. Build. Mater..

